# The First Case of Bridge-Enhanced Anterior Cruciate Ligament (ACL) Repair (BEAR) Procedure in Mississippi

**DOI:** 10.7759/cureus.44218

**Published:** 2023-08-27

**Authors:** Azeem U Khan, Rida Aziz, Michael Reen, William Walker, Philip Myers

**Affiliations:** 1 Orthopedic Surgery, William Carey University College of Osteopathic Medicine, Hattiesburg, USA; 2 Orthopedics, Singing River Hospital, Gulfport, USA

**Keywords:** knee injuries, acl remnant, anterior cruciate ligament (acl) reconstruction, bridge, acl tear, acl repair

## Abstract

In the past, surgical treatment of anterior cruciate ligament (ACL) tears has mainly involved reconstruction using allografts and autografts. The relatively new FDA-approved bridge-enhanced ACL repair (BEAR) procedure allows the body to use its innate healing properties to help repair the ACL using an absorbable protein-based implant. The procedure is currently being offered by surgeons in 44 states. This case describes the first BEAR procedure performed in the state of Mississippi.

A 47-year-old female of normal BMI presented to the orthopedic clinic with a chief complaint of right knee pain. The patient stated that she felt unstable on the injured knee, and the patient had positive anterior drawer and Lachman's tests on physical examination. MRI of the knee one month after injury revealed full-thickness ACL rupture. The patient underwent arthroscopic bridge-enhanced ACL repair in the right knee 43 days after the initial injury. The patient reported positive progress in her healing process at her three-month follow-up, and MRI at the three-month follow-up showed successful repair of the patient’s ACL. At six months post-operatively, the patient reported that she is still doing well, and she feels that the stability of her right knee has improved.

This case highlights an early trend towards repairs instead of reconstructions in ACL injuries for candidates that meet the following requirements: within 50 days of injury and have an intact tibial stump as recommended by the implant manufacturers.

## Introduction

In the past, surgical treatment of anterior cruciate ligament (ACL) tears has involved reconstruction using either autografts, allografts, or synthetic grafts [1]. The reconstruction technique depended on patient characteristics, such as age and functional demand. Primary repair was explored in the 1970s and 1980s but was not popularized due to the re-tear rate over two years being nearly 50% [2,3]. However, ACL repair is being re-popularized due to the recently FDA-approved bridge-enhanced ACL repair (BEAR) technique which has demonstrated safe and effective outcomes in randomized controlled trials, and the first BEAR procedure was performed on a human in 2015 [2,3]. The BEAR procedure involves an absorbable protein-based implant to be sutured to the torn end of the ACL which is then saturated with the patient's blood. This allows the ACL to heal using the implant as a bridge between the remaining ACL tissue, with the body absorbing the implant over time [4]. Currently, the procedure is being performed only in the United States with surgeons in 44 states currently offering the procedure. This case describes the first BEAR procedure performed in the state of Mississippi.

## Case presentation

A 47-year-old female with a normal BMI presented to the orthopedic clinic with a chief complaint of right knee pain. She states that the injury was caused by falling off a hoverboard a few weeks prior to the presentation. She described the pain as dull and achy and stated that she felt unstable on the injured knee. On physical examination, the patient had positive anterior drawer and Lachman's tests.

**Figure 1:**
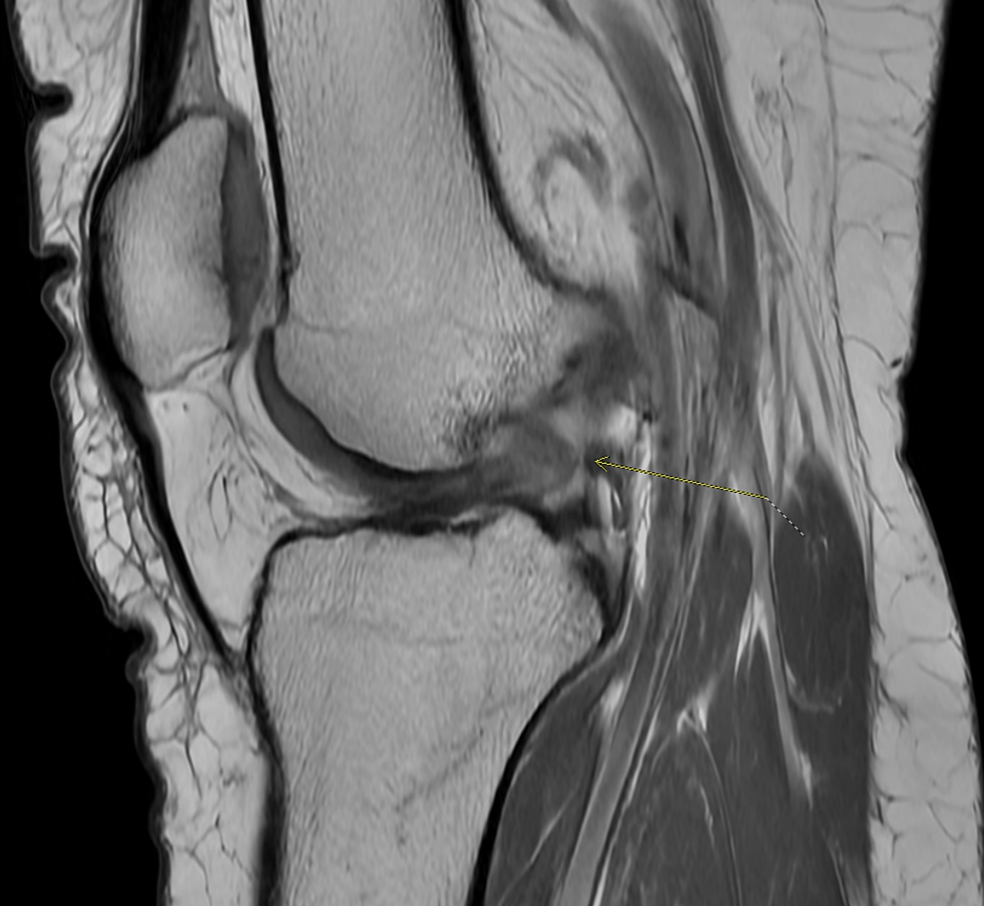
Sagittal MRI of the patient's right knee demonstrating ACL rupture. ACL: anterior cruciate ligament

An ACL tear was suspected, and the patient was sent for MRI imaging. The MRI of the right knee one month after the injury revealed a full-thickness ACL tear with an intact tibial stump of the remaining ACL tissue (Figure 1). The patient was educated and counseled on conservative as well as surgical options including both reconstruction and repair. After reviewing her treatment options and careful consideration, the patient expressed her desire for an arthroscopic bridge-enhanced ACL repair (BEAR).

During the arthroscopic procedure which was performed 43 days after the injury, both the lateral and medial compartments were visualized with no indications of medial or lateral meniscal tearing. A full-thickness ACL tear was appreciated with an intact tibial stump. The BEAR implant was placed with appropriate tension and tunnel placement arthroscopically utilizing the tibial stump. The implant was then saturated with 10 cubic centimeters (cc) of the patient’s blood intra-operatively to promote clot formation. The remaining surgery proceeded as usual with the patient being placed in a T Scope knee brace locked in full extension.

The patient was instructed pre-operatively to use crutches and keep the knee brace locked in extension while walking for the first four weeks as well as during sleep for the first six weeks. The patient was also informed that she would not be cleared to drive until she was able to fully bear weight on her right knee without crutches, and she could achieve 60° of flexion as well. Patient was also instructed to perform at-home physical therapy exercises focused on the quadriceps muscles, patellar mobilization, and active range of motion (ROM) with the brace being locked at 0°-45° of flexion. Passive ROM in flexion was not performed at any point during physical therapy.

On the initial two-week follow-up, the patient reported no adverse events and stated her recovery was going well. She began outpatient physical therapy two to three times every week, and her brace range was now set at 0°-90° of flexion while seated and during physical therapy. At four weeks, she began to wean from her crutches, and she no longer needed her brace to be locked in extension during sleep at six weeks. She was able to change to a functional knee brace from her t-scope brace at eight weeks as her active ROM had increased to over 110° of flexion. Physical therapy began to focus on hamstring muscles and proximal hip muscles strengthening in addition to the quadriceps strengthening exercises already being performed.

**Figure 2:**
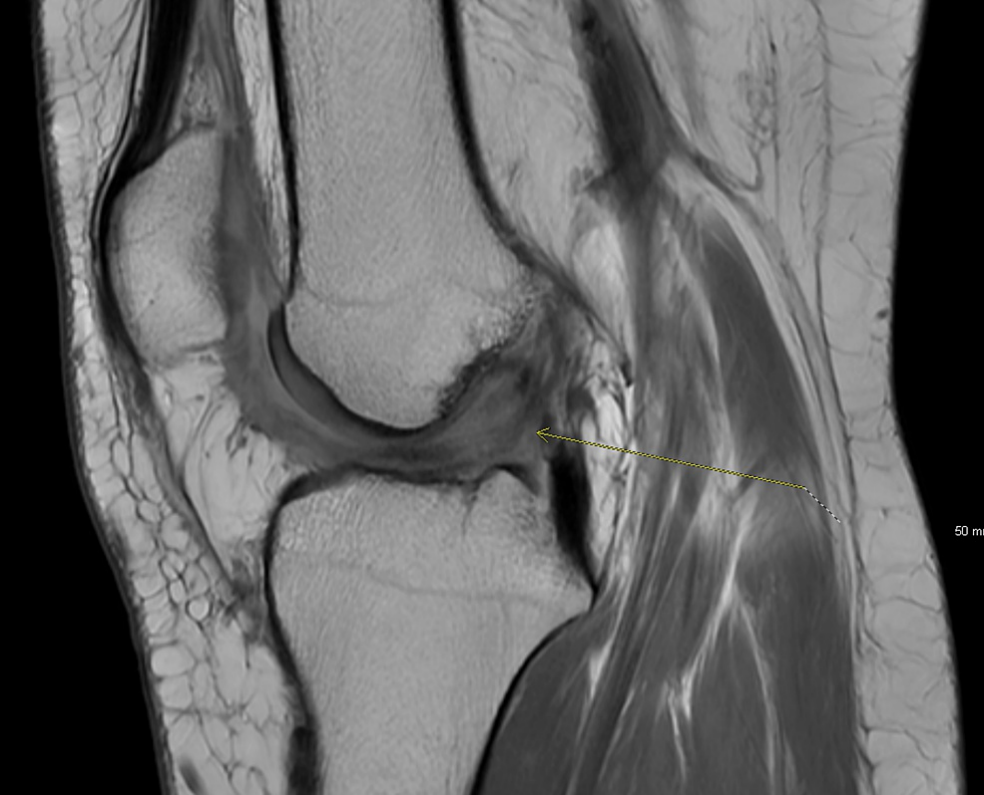
Sagittal MRI of the patient's right knee three months post-operatively demonstrating expected post-surgical changes following BEAR procedure. BEAR: bridge-enhanced ACL repair; ACL: anterior cruciate ligament

At her three-month follow-up, the patient stated that she felt much more stable on her leg during ambulation and felt her physical therapy was proceeding well. MRI imaging on three-month follow-up demonstrated successful repair of the ACL with expected post-surgical changes (Figure 2). The patient was instructed to continue physical therapy during which she continued to work on returning muscle strength and increasing active ROM. At six months post-operatively, the patient reports increased right leg strength with no pain during activity. The patient also reported that her active ROM is now equal in bilateral knees.

## Discussion

The patient presented had a successful repair of her ACL using the BEAR procedure. Most recent studies suggest that re-tear rates for ACL repairs with the BEAR technique are similar to the re-tear rates with ACL reconstruction [5]. This demonstrates superiority to the ACL repairs performed prior to the development of the BEAR technique [2,5]. Additionally, patients seem to physically recover at a similar rate of approximately nine months. However, there is evidence that those patients are more confident about returning to sports sooner post-operatively who underwent ACL repair using the BEAR procedure in comparison to patients who underwent reconstruction [6].

While the BEAR technique seems to have comparable outcomes to autograft and allograft, only certain patients will qualify for the BEAR technique [5,7]. While some studies of the BEAR procedure have chosen to include only patients who are within 45 days of injury, the implant manufacturer currently recommends that candidates for the BEAR procedure must have injured their ACL within 50 days of the procedure and they must have an intact tibial stump for the attachment of the implant [5,8,9]. ACL reconstructions in comparison do not have such qualifications necessary for operation. This creates timing and anatomical constraints for the BEAR technique.

Studies have shown that patients who have undergone repair with the BEAR technique demonstrated superior hamstring strength in comparison to autograft reconstruction [5,7]. The superior hamstring strength post-operatively demonstrates an advantage of the BEAR procedure to ACL reconstruction. Although trials of 10-15 years on humans have not been completed to assess post-traumatic osteoarthritis rates with this procedure, porcine models have demonstrated decreased macroscopic cartilage damage in pigs receiving the BEAR procedure in comparison to pigs that received bioenhanced ACL reconstruction [10]. This evidence may suggest that the BEAR procedure may contribute to joint preservation when compared to ACL reconstruction.

## Conclusions

This study demonstrates the successful short-term outcome of a patient undergoing the BEAR procedure. Long-term data continues to be collected for the BEAR procedure. This case highlights an early trend towards repairs instead of reconstructions in ACL injuries for candidates that meet the requirements (within 50 days of injury and have an intact tibial stump). Long-term studies will ultimately determine if the BEAR procedure shows superiority. 
